# Downscaled and debiased climate simulations for North America from 21,000 years ago to 2100AD

**DOI:** 10.1038/sdata.2016.48

**Published:** 2016-07-05

**Authors:** David J. Lorenz, Diego Nieto-Lugilde, Jessica L. Blois, Matthew C. Fitzpatrick, John W. Williams

**Affiliations:** 1 Center for Climatic Research, University of Wisconsin-Madison, Madison, Wisconsin 53706, USA; 2 University of Maryland Center for Environmental Science, Appalachian Lab, Frostburg, Maryland 21532, USA; 3 School of Natural Sciences, University of California, Merced, California 95343, USA; 4 Department of Geography, University of Wisconsin-Madison, Madison, Wisconsin 53706, USA

**Keywords:** Palaeoclimate, Palaeoecology, Climate-change ecology, Biogeography

## Abstract

Increasingly, ecological modellers are integrating paleodata with future projections to understand climate-driven biodiversity dynamics from the past through the current century. Climate simulations from earth system models are necessary to this effort, but must be debiased and downscaled before they can be used by ecological models. Downscaling methods and observational baselines vary among researchers, which produces confounding biases among downscaled climate simulations. We present unified datasets of debiased and downscaled climate simulations for North America from 21 ka BP to 2100AD, at 0.5° spatial resolution. Temporal resolution is decadal averages of monthly data until 1950AD, average climates for 1950–2005 AD, and monthly data from 2010 to 2100AD, with decadal averages also provided. This downscaling includes two transient paleoclimatic simulations and 12 climate models for the IPCC AR5 (CMIP5) historical (1850–2005), RCP4.5, and RCP8.5 21st-century scenarios. Climate variables include primary variables and derived bioclimatic variables. These datasets provide a common set of climate simulations suitable for seamlessly modelling the effects of past and future climate change on species distributions and diversity.

## Background & Summary

A productive new synthesis is being forged among ecologists, conservation biologists, biogeographers, and paleoecologists^1–7^, motivated by a common goal of understanding how climate change has affected and will affect the diversity and distribution of species. Species extinction rates currently are 100 times background rates^8^ and climate change is expected to further stress threatened populations^9–12^. Metaanalyses suggest that 8% of species will go extinct if global temperatures rise above 3 °C (ref. 11). Given these projections, there is an urgent need to understand how environmental conditions govern the distribution and abundance of organisms, and to apply this knowledge to help species adapt to climate change.

Within this synthesis, the geohistorical record—with many well-documented instances of past climate changes and biological responses—is essential^5,9,10,13,14^. Most notably, the glacial-interglacial cycles of the last million years provide the closest equivalent to the rates and magnitudes of climate change expected for this century^13,15^. Ecological research opportunities created by these periods of transition include understanding (1) the stability of ecological niches during periods of environmental change^16–18^, (2) rates of biological response to abrupt climate change^19–23^, (3) the linkage between no-analogue communities and no-analogue climates^13,24–26^; (4) the predictive ability of ecological forecasting models and their underlying assumptions^27,28^; (5) the influence of past climate changes and human activity on current genetic diversity^29,30^; (6) glacial refugia, areas of climate stability, and other mechanisms by which species persisted during past adverse climates^31–33^; and (7) the causes and consequences of the late-Quaternary extinctions of large-bodied vertebrates^34,35^.

However, despite calls for the full synthesis of contemporary observations, geohistorical data, experiments, and 21st-century projections to build an integrated science of climate change and biodiversity assessment^6,36,37^, progress has been limited by the scarcity of climate simulations that seamlessly extend from the past into the future. As a result, future- and paleo-oriented studies of biodiversity dynamics generally remain divided, with few directly integrating the fossil record with future projections^38^.

Because Earth system models (ESMs) are computationally expensive, most simulations are run for limited time windows (decades to centuries) according to prescribed experimental protocols^39,40^. Common modelling targets include the middle Pliocene (3.3 to 3.0 million years ago), last interglacial (125,000 years ago), last glacial maximum (21,000 years ago), mid-Holocene (6,000 years ago), and last millennium^39^. Some ESM’s are run for longer periods to assess the evolving response of the earth system to transient forcings, e.g., to shifting orbital variations, rising greenhouse gases, and meltwater pulses during the last 21,000 years^41,42^, or to variations in solar luminosity, volcanic eruptions, orbital variations, greenhouse gases, and land cover over the last millennium^43,44^.

ESM simulations usually have systematic biases compared to observations and their native spatial resolutions often are too coarse for biological modelling. Simply using their simulations without a common debiasing and downscaling approach will lead to systematic biases among the past, present, and future climate simulations, deeply confounding any ecological inferences based on these simulations. Moreover, ensembles comprising multiple ESMs generally fit better to observations than individual ESMs^45^. Hence, an ecologist seeking to model the effects of past, present, and future climate change on biodiversity is forced to stitch together climate simulations from many models, a labour-intensive process that often results in unnecessary duplication of efforts among research teams.

Here we present debiased and downscaled climate simulations for North America, from 21,000 years ago to 2100AD (Data Citation 1), using a common set of observational datasets and methods. The paleoclimatic datasets are based on transient simulations from two ESMs^41,46,47^, while the 20th- and 21st-century simulations are based on 12 ESMs using the CMIP5 historical, RCP4.5, and RCP8.5 scenarios^40^. These downscaled datasets include standard indices of monthly temperature and precipitation, other climate variables useful to modelling surface energy and moisture budgets, and derived variables such as growing degree days and actual and potential evapotranspiration. All variables are available as decadal averages of monthly values for the last 21,000 years, and as monthly values from 1950AD to 2100AD. From the paleoclimatic simulations, we extract century-scale averages and other statistics, spaced 500 years apart, while from the 21st-century simulations we extract decadal-scale averages, spaced a decade apart. Note that this debiasing and downscaling does not guarantee that the ESM simulations are accurate predictors of past and future climates, merely that they have been standardized to be consistent with each other and with contemporary observational data. As always, ESMs and their simulations should be used carefully and critically, and paleoclimatic simulations should be checked for consistency with paleoclimatic proxy data, which, of course, carry their own uncertainties.

## Methods

### Overview

**Figure 1:**
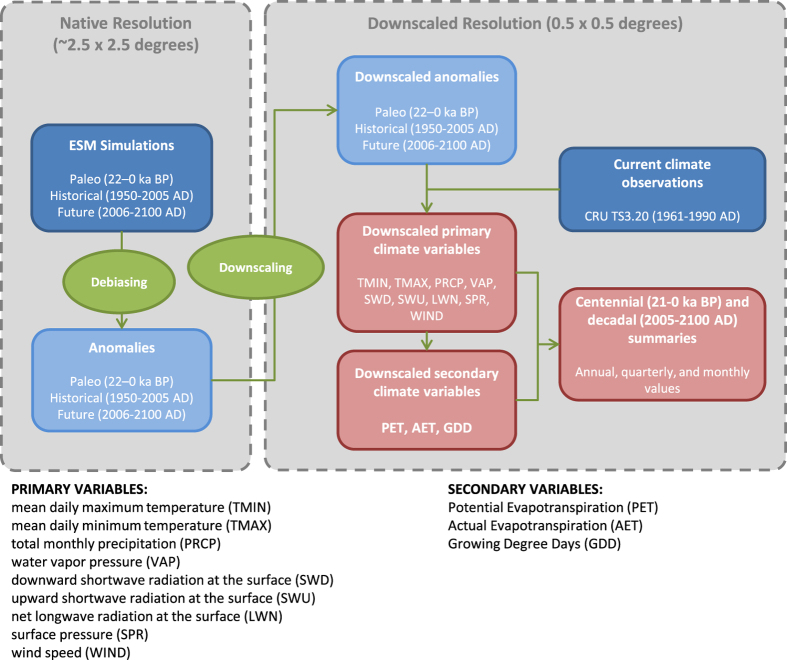
Workflow diagram summarizing the major steps used to generate the debiased and downscaled paleoclimate and 21st-century datasets described here. The primary climate variables are first debiased by differencing (or similar calculation, see Section 3) each paleoclimate or future climate simulation from a climate simulation representing present climates. These anomalies (also known as factors) are then downscaled via bilinear interpolation to a higher-resolution grid corresponding to the resolution of the modern observational dataset. The anomalies are then added to (or multiplied by, see Section 3) the observational data to produce the downscaled primary variables. From these downscaled primary variables, secondary bioclimatic variables (GDD, AET, PET) are calculated. Finally, statistical summaries of all variables are calculated at centennial (paleoclimatic simulations) and decadal resolution (21st-century projections).

Statistical downscaling and debiasing followed a multi-step approach (Fig. 1). First, most of the primary climate variables were debiased and downscaled using the standard change-factor approach^48^ applied separately to each month, or to each season if monthly data are unavailable. Climate variables archived only as seasonal values then were interpolated to monthly values. Primary climate variables are mean daily maximum temperature, mean daily minimum temperature, total monthly precipitation, water vapour pressure, downward and upward shortwave radiation at the surface, net longwave radiation at the surface, and wind speed (Fig. 1, Table 1 (available online only)). From these primary variables, a secondary set of climate variables were calculated for each month: potential and actual evapotranspiration (PET and AET) and growing degree days (base 0° and 5 °C; GDD0 and GDD5). For the paleoclimatic simulations, some grid cells were land in the past but are under water today due to ice sheet melting and sea level rise during the last deglaciation. For these grid cells, paleoclimates were inferred by spatial extrapolation.

For the paleoclimate simulations, two climate models were downscaled: CCSM3 (refs 41,46) and ECBilt-CLIO^47^. For the 21st-century climate simulations, 12 models from the CMIP5 model archive were downscaled (Table 2), using the historical, RCP4.5, and RCP8.5 scenarios.

The spatial domain is North America (48° to 173°W, 10° to 80°N) and the spatial resolution of the downscaled climate simulations is 0.5×0.5 degrees. The temporal domain for the paleoclimate simulations is the last glacial maximum to the 20th century (CCSM3: 22,000 years before 1950AD (ka BP) to 1990AD, ECBilt: 21 ka BP to 1950AD). The temporal domain for the 21st century climate simulations is 1950AD to 2100AD. The temporal resolution of the downscaled paleoclimate simulations is decadal means for each calendar month, while the temporal resolution of the 21st-century climate simulations is monthly data for each year. This temporal resolution is determined by the archived climatic simulations. The native temporal resolution of atmospheric circulation models typically is subhourly, but not all time steps are publicly archived, so part of the downscaling of the paleoclimatic simulations involved inferring monthly values from archived seasonal means.

After initial downscaling, paleoclimate datasets were prepared for biogeographic hindcasting by extracting century-scale bins of the data spaced 500 years apart (each bin was 200 years thick and centered on the 500 year time step), from 21 ka BP to the 20th century. For each century-scale bin, annual and seasonal averages and other statistical summaries of climate variables were calculated from the decadal mean monthly data in the 100 years before and after the time step, and values were averaged across the 20 decades (Table 3). For the 21st-century climate simulations, 20-year monthly averages and other statistical summaries were extracted at 10-year time intervals (for 2011–2030, 2021–2040, etc.). All steps are described in more detail below. The centennial and decadal summaries are saved as geotiffs (Table 1 (available online only)).

### Data sources

#### Observational climate datasets for the 20th and 21st centuries

The change factor approach requires contemporary observational datasets against which the ESM simulations are debiased and downscaled^48^. Monthly precipitation, maximum and minimum daily temperature, and vapour pressure (1901–2011) are from the Climate Research Unit (CRU) TS v.3.20 dataset^49^. Wind speed is from a 12 month climatological CRU dataset for the time period 1961–1990 (ref. 50). The spatial resolution of all CRU data is 0.5×0.5 degrees. The radiation data are from the NASA/GEWEX Surface Radiation Budget (SRB) Release-3.0 dataset^51,52^. The GEWEX radiation data are interpolated from a 1×1 degree grid to the CRU grid using bilinear interpolation. For the GEWEX shortwave and longwave radiation, the monthly anomalies from the climatology are considered significantly less reliable than the average climatology itself, and therefore only the climatology is used, for 1983–2007. The top-of-atmosphere solar radiation from 21 ka BP to present is calculated using the algorithm of Berger^53^. Surface pressure in the present is estimated from elevation data using guidelines recommended by Allen *et al.*^54^. The elevation data are from the Global Land One-km Base Elevation Project (GLOBE)^55^. The elevation is averaged spatially over the CRU grid boxes to create a lower resolution elevation dataset consistent with the other data. We estimate past surface pressure from the dataset of land and ice elevation of Peltier^56^.

To maximize uniformity among downscaled variables and models, we sought to use a common time period for the observational time period. However, perfect uniformity was impossible because of differences among model simulations in their 20th century start/end dates and differences among observational datasets in their temporal extent. For temperature, precipitation, and vapour pressure, the time period used for the baseline climate was 1901 to 2011 for the paleoclimatic simulations and 1950 to 2005 for the CMIP5 simulations. For wind speed, the baseline time period is 1961–1990 for all downscaling analyses. For shortwave and longwave radiation, the baseline time period is 1983–2007.

#### Climate models

The paleoclimate simulations consist of a 22,000 year transient simulation from the Community Climate System Model (CCSM3)^41,46^ and a 21,000 year transient simulation using ECBilt-CLIO^47^. Decadal means of paleoclimatic simulations were obtained from CCSM3 (Feng He, pers. comm.) and EC-BILT (http://apdrc.soest.hawaii.edu/datadoc/sim2bl.php). CCSM3 was forced by prescribed trends in orbital parameters, ice sheet extent and height, sea level, greenhouse gases, and meltwater pulses to the North Atlantic, while ECBilt-CLIO was forced by prescribed trends in orbital parameters, ice sheet extent and height, and greenhouse gases. Often, when using output from climate models, all simulations are given equal weight in derived ensembles. However, the ECBilt-CLIO simulation carries known simplifications in its model structure relative to the CCSM3 simulation, including: (1) a quasi-geostrophic atmospheric model that constrains vertical static stability; (2) 3 vertical levels versus 26 for CCSM3; (3) a radiation code linearized about present day conditions, and (4) prescribed seasonally and spatially varying cloud cover climatology. Therefore, we recommend that any use of these simulations place greater weight on the CCSM3 simulations. Note too that the ECBilt-CLIO archive did not include vapour pressure and wind speed, so these variables are not downscaled for this model.

Output from twelve climate models were downloaded from the Program for Climate Model Diagnosis and Intercomparison (PCMDI) website (http://cmip-pcmdi.llnl.gov/cmip5/data_portal.html), for the historical, RCP4.5, and RCP8.5 scenarios, as reported in the Fifth Assessment Report of the Intergovernmental Panel on Climate Change (IPCC AR5). One model was chosen from each modelling centre and models were selected only if they archived all climate variables used for the analyses here. Among IPCC models, the quality of the simulations vary and, depending on the variable, some models are more skilful than others, with no clear signal of some models consistently outperforming others. Therefore, it is generally understood that the projections of all models should be considered with equal weight. Note, however, that two of the models (ACCESS1.3 and MIROC5) do not conserve atmospheric water mass^57^. Analyses relying on these individual models should be aware of the in-built hydrological imbalances and the associated biases in modelled radiative forcing. Nevertheless, skill tests show that mean climates from model ensembles consistently outperform all individual models in nearly every respect^58^.

### Debiasing and downscaling of primary variables from paleoclimatic simulations

#### Overview

**Figure 2:**
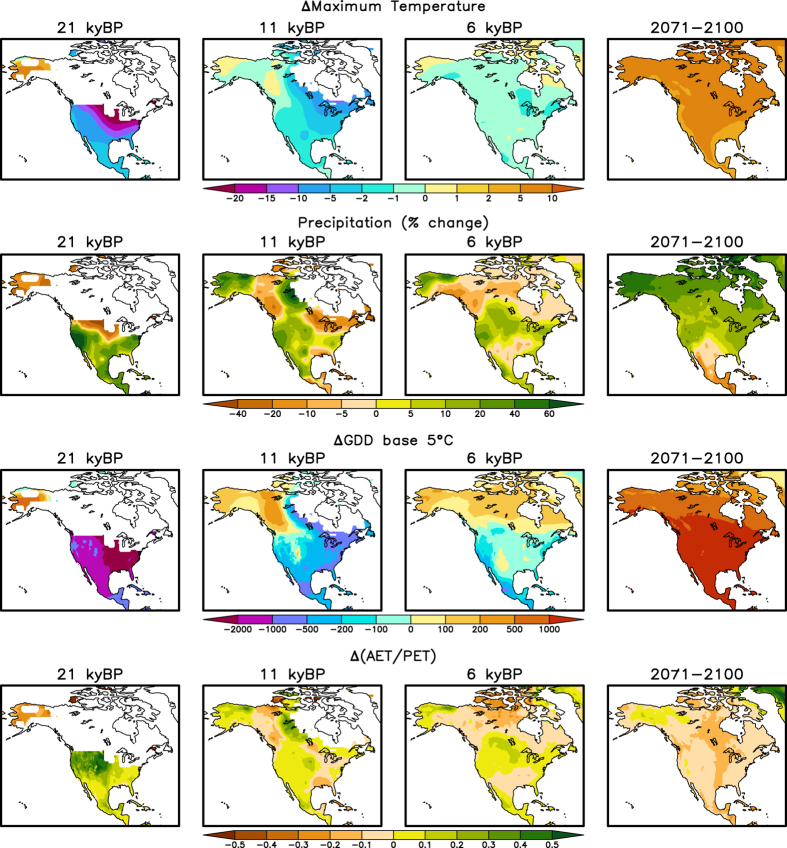
Anomaly maps showing examples of the downscaled differences between simulated past and future climates to the 20th century baselines. Top row: difference maps for mean maximum daily temperature, for 21 ka BP, 11 ka BP, 6 ka BP and 2071–2100AD. The paleosimulations are for the downscaled SynTrace CCSM3 simulation, with locations under the ice sheet masked out. The upper-right plot is the difference between 2071–2100 and 1950–2005, averaged over 12 climate models and for the RCP8.5 scenario. Second row: as top row, but for the percent change in precipitation compared to present. Third row: as top row, but for growing degree days (GDD) base 5C. Fourth row: as top row, but for the ratio of mean annual AET to mean annual PET.

To debias the variables, we calculated the difference between the modelled paleoclimate and the modelled present climate (Fig. 1). The resulting anomaly was then downscaled through bilinear interpolation to the higher resolution of the observational climatology to produce the debiased and downscaled paleoclimate at higher resolution (Fig. 2). This differencing removes any systematic difference between modeled and simulated climates as long as that bias is constant through time^59^. For example, for temperature and net longwave radiation at the surface, we calculated the difference between the modelled variable and the modelled present state of the climate variable (defined for this step as the last 110 years of the paleoclimatic model run) at each grid cell. This anomaly was then bilinearly interpolated to the 0.5° grid of the CRU TS 3.20 dataset. For climate variables bounded by zero, such as wind speed and water vapour pressure, we employed the factor approach, i.e., the ratio between the modelled paleoclimate and the modelled present climate was calculated, interpolated, and then multiplied by the observed climatology. Some variables required special handling during debiasing and downscaling, which we describe further below.

#### Shortwave radiation

A significant portion of the change in surface shortwave radiation over the past 21,000 years is not related to the internal physics of the climate models but is instead externally forced by changes in the earth’s orbit. Therefore when calculating shortwave anomalies, we normalize both the upward and downward solar radiation at the surface by the maximum top of atmosphere downward solar radiation: $\hat{S}=S/B$, where *S* is either the raw downward or raw upward solar radiation at the surface, *B* is the top-of-atmosphere solar radiation which is a function of latitude and the earth’s orbital parameters at time *t*^53^, and $\hat{S}$ is the normalized downward or upward solar radiation. $\hat{S}$ must fall between 0 and 1. To automatically preserve this 0 to 1 range, we express the value of $\hat{S}$ in an alternate climate $\left({\hat{S}}_{\mathrm{alt}}\right)$ as the present climate $\left({\hat{S}}_{0}\right)$ raised to a positive power, γ:

(1)Sˆalt=Sˆ0γ

To use equation 1 for downscaling, we solve for γ:

(2)γ=log(Sˆalt)/log(Sˆ0)

where both $\hat{S}$’s are the low-resolution climate model values. The low-resolution γ is then interpolated to the 0.5° grid of the observed climate data. Finally the high resolution γ is used to transform the present climate ${\hat{S}}_{0}$ using equation 1. This approach is analogous to the traditional change-factor approach. The only difference is that the transformation is modified in order to rescale modeled solar radiation to the range of values allowable given past orbital configurations.

#### Precipitation

For precipitation, which is bounded by zero, the factor method described above is a commonly used downscaling approach. Unfortunately, locations that are very dry tend to have very large fractional changes in precipitation and vice versa. This creates a problem for regions that are very dry in the climate model but very wet in observations. In a dry region, the climate model might predict a factor-of-10 increase in precipitation. While this is reasonable in a desert, it almost certainly artificial in a wet region. Therefore it is unrealistic to assume that the factor to multiply the observed climatological precipitation can be taken directly from the climate model.

To avoid the above issue, we use a method based on quantile mapping^60^. Quantile mapping uses the fact that the precipitation variability in dry regions is also large compared to wet regions. The historical relationship between the variability in the climate model and the observational dataset is used to map the simulated change in precipitation to a corresponding change in observations. The details are as follows: consider a 100-year record of July precipitation in observations and in a climate model, where the observations are first averaged in space to the low-resolution climate model grid. Both the observations and the climate model time series are then independently sorted from the smallest July precipitation to the largest July precipitation. Let the sorted observations and climate model precipitation be denoted as *p_j_* and *q_j_*, respectively. The empirical, monotonic function, *p_j_*=*f*(*q_j_*), describes a mapping from modelled precipitation to observations that transforms the modelled probability density function (PDF) to the observed PDF. To use the function, *f*, for past or future modelled precipitation (*q_alt_*), we use linear interpolation to estimate *f* at points between the *q_j_*’s and at points beyond the range of ‘observed’ climate model precipitation (i.e., less than *q_1_* or greater than *q*_100_). The resulting (low resolution) observed precipitation estimate, *p_alt_*=*f*(*q_alt_*), is then normalized by the low-resolution climatology and, like the factor approach, the resulting factors are then interpolated to the high-resolution observational grid. The high-resolution observed climatology is then multiplied by the high-resolution factors to make the final downscaled precipitation.

The above methodology is essentially the standard quantile mapping approach of Wood *et al.*^60^ and is also used to downscale the precipitation data for the future climate simulations. We modify the method for the paleoclimatic downscaling because we are limited to decadal data, which means we have only 11 decades of observations over the 20th and early 21st centuries. Because the limited sample can lead to noise in the function, *f*, we instead use the 11 observations of *p_j_* and *q_j_* to fit a linear least-squares line (*p*=*aq*+*b*) and we use the line as the function giving *p_j_* as a function of *q_j_*. As in the standard case, potential issues arise when one has values of *q*_*alt*_ that are beyond the range of ‘observed’ climate model precipitation. In our case, we use the above linear fit when *b*≥0 or when *q*_*alt*_≥*q*_1_. When *b<*0 and *q*_*alt*_<*q*_1_, on the other hand, *p*=*q* (*aq*_1_+*b*)*/q*_1_. This solution is continuous with *p*=*aq*+*b* at *q*=*q*_1_ and it automatically satisfies the constraint that *p>*0 when *q>*0.

### Interpolating seasonal to monthly variables

At the time of analysis, many of the variables for CCSM3 and all of the variables for ECBilt-CLIO were available only as seasonal means. We interpolated these variables to monthly values, both for consistency and for the calculation of potential and actual evapotranspiration, which benefit from having monthly resolved variables. However, linearly interpolating seasonal data to monthly values strongly dampens the annual cycle and moreover produces monthly data that is usually not consistent with the seasonal data from which it is derived (e.g., summer temperature is not equal to the average of the June, July and August temperature in the derived monthly data, see examples in technical validation section). Here we develop a method for creating consistent monthly data from seasonal data.

#### Temperature and other anomaly variables

The simplest case is for (maximum and minimum) temperature where, for all practical purposes, there is no lower or upper bound on allowable values. Let *S*1, *S*2, *S*3 and *S*4 be the seasonal mean temperature for winter, spring, summer and fall, respectively. Let *T_j_* (*j*=1,12) be the monthly mean temperature for the 12 calendar months, which we want to estimate from *S_j_*. The *T_j_* must satisfy the following constraints:

(3)T1+T2+T12=3S1T3+T4+T5=3S2T6+T7+T8=3S3T9+T10+T11=3S4.

The specification of *T_j_* is obviously under-constrained. One solution is to let monthly temperature be equal to the corresponding seasonal temperature (i.e., T_1_=T_2_=T_12_=S_1_, etc.). This solution is highly unlikely because it produces an annual cycle that is not very smooth. We hypothesize that the most likely solution is the ‘smoothest’ solution that also satisfies equation 3. To quantify smoothness we use the simple second order approximation of the second derivative: T_j−1_−2T_j_+T_j+1_. When the second derivative is small the ‘smoothness’ is high, so we want to minimize:

(4)min(12∑j=112(Tj−1−2Tj+Tj+1)2)

where *T*_*0*_ is December temperature and *T*_*13*_ is January temperature. We solve equations 3 and 4 using Lagrange multipliers, which leads to a linear 16×16 matrix equation. For the downscaling, this procedure is applied to the debiased low-resolution anomalies.

In summary, the steps are: 1) calculate the difference between the modelled seasonal temperature and the modelled current seasonal temperature, 2) convert the seasonal anomalies to monthly anomalies using the above procedure, 3) interpolate the monthly anomalies to the high resolution grid, and 4) add the resulting high resolution monthly anomalies to the observed climatology. The same procedure is used for the net longwave radiation at the surface. See the Technical Validation section for an assessment of this method for converting seasonal variables to monthly variables.

#### Precipitation and other factor variables

For precipitation the method is more complicated because one must ensure that all monthly precipitation amounts are at least zero. We solve this problem by minimizing the second derivative of the logarithm of the precipitation:

(5)min(12∑j=112(log(Pj−1)−2log(Pj)+log(Pj+1))2)

where *P_j_* is the estimated monthly precipitation and, as before, *P*_0_ is December precipitation and *P*_13_ is January precipitation. The constraints are exactly analogous to equation 3 for temperature. Using Lagrange multipliers, the solution can be represented by a system of 16 equations, some of which are nonlinear. We solve the system of equations using MINPACK^61^, which solves a system of nonlinear equations using a modification of the Powell hybrid method. See the Technical Validation section for an assessment of this method for converting seasonal variables to monthly variables.

For downscaling past precipitation, we represent past precipitation as a fraction (*f*) of the present precipitation (i.e., *P*_past_=*f(P*_present_)). Therefore, the above method for the mean precipitation needs to be modified. Let *P_j_* be the observed 12 month climatology of precipitation in the present, *f_j_* be the monthly fraction of present precipitation that we want to calculate, and *F_j_* be the seasonal fraction of present precipitation from the climate model (where *j*=1,4 corresponds to winter, spring, summer and fall, respectively). The four constraints are:

(6)f1P1+f2P2+f12P12=F1(P1+P2+P12)f3P3+f4P4+f5P5=F2(P3+P4+P5)f6P6+f7P7+f8P8=F3(P6+P7+P8)f9P9+f10P10+f11P11=F4(P9+P10+P11).

The function we want to minimize subject to the above constraints is:

(7)min(12∑j=112(log(fj−1)−2log(fj)+log(fj+1))2)

The same method is used for water vapour pressure and wind speed.

#### Shortwave radiation

For shortwave radiation, we simply apply the temperature equations (3 and 4) to the power (γ) except that we first take the logarithm of the seasonal power, to help satisfy the constraint of a positive power. Once the monthly log(γ) is found we then transform back to the raw powers, γ.

### Secondary variables

After all primary variables are debiased, downscaled, and transformed to monthly resolution, they are used to derive the secondary variables as described below.

#### Potential evapotranspiration

We estimate the potential (reference) evapotranspiration using the Penman-Monteith method of Dobrowski *et al.*^62^ Their method is very similar to the standard defined in Allen *et al.*^54^, but they incorporated several modifications to create a reference that behaves more realistically under cold and snowy conditions. Our only modifications from Dobrowski *et al.*^62^ are (1) the use of downscaled albedo (specifically, upward shortwave radiation at the surface) instead of one or two predetermined values depending on the presence of snow cover, and (2) we did not estimate the effect of fine-scale local topography on surface radiation.

#### Actual evapotranspiration

Estimating actual evapotranspiration requires an estimate of soil moisture. We used the ‘single-bucket’ methodology of Lutz *et al.*^63^ to model actual evapotranspiration (AET) but with modifications to the snowmelt model, as described elsewhere^62^. We use a constant soil water holding capacity of 150 mm. Estimates of actual evapotranspiration are not particularly sensitive to reasonable alternative water holding capacities. For example, re-running the model using observed soil water holding capacity, Dunne and Willmott^64^ demonstrated that constant water capacity leads to a domain-averaged error in the annual average AET of only 3.8%. Furthermore, given that soil water holding capacity is not constant over millennia, we thought it was best to simply assume a constant capacity rather than assume the present water-holding capacity applies to the past.

#### Growing degree days

With daily temperature data, the calculation of Growing Degree Days (GDD) is straightforward. Unfortunately, we only have monthly temperature (*T*¯). If we make some assumptions about the shape of the probability density function (PDF) for daily temperature variability, however, we can calculate GDD given daily temperature variance. For each day, the GDD base *T*_*0*_ is defined as:

(8)GDD=max(T−T0,0)

where T is the ‘average’ daily temperature (*T*=(*T*_max_ *−T*_min_) */*2). If we know the form of the daily temperature PDF (here referred to as *f* (*T* )), then the climatological GDD is:

(9)GDD=∫−∞∞f(T)max(T−T0,0)dT=∫T0∞f(T)(T−T0)dT

If *f*(*T*) is a normal distribution with mean *T*¯ and standard deviation σ, then equation 9 can be solved to give a relationship between *GDD*, *T*¯ and σ, given *T*_0_:

(10)GDD=σ2πexp(−12(T¯−T0σ)2)+T¯−T02erfc(T0−T¯2σ)

where *erfc* is the complementary error function. We test the assumptions used to derive equation 10 by analyzing all daily NOAA COOP weather stations with fewer than 20 missing days from 1950 to 2009 (=108 stations). For each station we calculate the actual average monthly GDD (for base 0 °C and 5 °C) as well as the monthly mean temperature and the monthly mean of daily temperature standard deviation. From the monthly mean and the monthly mean daily standard deviation, we estimate GDD using equation 10. We also apply equation (8) directly to the monthly mean temperature, which is the usual way GDD is calculated from monthly data. See the Technical Validation section for an assessment of the GDD estimator.

To use equation 10, we need the daily standard deviation $\sigma_{daily}^{2}$. For this project we estimate the daily standard deviation from the monthly standard deviation using the same daily COOP stations. If the daily temperature is uncorrelated in time (i.e., white noise) then the relationship between the daily and monthly precipitation is:

(11)σ2=nσmonthly2

where *n* is the number of days in the month. In reality, temperature autocorrelation is >0 so that the actual monthly σ is greater than that given by equation 11. Some of the extra auto-correlation or ‘memory’ comes from internal ‘weather’ variability and some comes from the annual cycle (e.g., after removing the monthly mean in April, there is still an upward trend in temperature (on average) that manifests itself as a positive auto-correlation). The memory due to the annual cycle is calculated by assuming the daily annual cycle is a piecewise linear function based on the climatological monthly-mean annual cycle, where the daily-mean cycle is precisely equal to the monthly mean at the center of each month. Next, after removing the monthly mean, we calculate the standard deviation of the daily annual cycle over each individual month. The formula for the daily standard deviation due to the annual cycle at month *j* (=1, 12) is:

(12)σannual=nj5a+2+6a+a−+5am2192

where *n_j_* is the number of days in month *j*, *a*_+_=2(*T*_*j*+1_ *− T_j_*)*/*(*n*_*j*+1_+*n_j_*) and *a_−_*=2(*T_j_−T*_*j*−1_)*/*(*n_j_*+*n*_*j*−1_). Here we assume that the extra memory from internal variability is the same for all stations, so that its effect can be estimated empirically by:

(13)σdaily2=anσmonthly2+σannual2

where *a* is the empirical constant. Equation 13 is fit using least squares (with no intercept), and the value of the constant *a* is 0.178.

Using equation 13, we calculate the daily standard deviation for each grid point in the CRU temperature data. We chose not to estimate the standard deviation for the past simulated temperatures, because we only have decadal means for temperature and the time-scales are very different from the daily observations available today. Instead, we apply the daily standard deviation from the modern data to the paleosimulations and assume that the daily standard deviation is constant across time. The mean temperatures for equation 10 are taken directly from the downscaled temperatures.

### Treatment of former land grid cells and ice-covered surfaces

Sea level rise has submerged portions of coastal North America that were above sea level during the last glacial period. To identify grid cells that were land in the past but are marine now, we used the paleoshoreline maps for North America developed by Patrick Bartlein at the University of Oregon, which are based on digitizing the topographic anomalies from Peltier^56^ and interpolating them onto a contemporary digital elevation model^65^. These paleoshoreline reconstructions are available at 1,000 year intervals. The digitized shapefiles were clipped and rasterized to the same spatial domain and resolution as the climatic data. Each decade was assigned to the temporally closest paleoshoreline (e.g., 1 ka BP paleoshorelines were assigned to decadal data from 0.5 ka BP to 1.5 ka BP). To estimate the past climates for these now-submerged grid cells, we extrapolate from nearby grid points that are land in the current climate. One approach would be to use the closest land grid point but this is more subject to local noise. Alternatively, one could average all land grid points within a certain radius of the target grid point. However, the optimal averaging radius depends on the distance to the closest land grid point. Instead we incrementally increase the ‘search radius’ and use the smallest radius that includes land grid points. In addition, because we expect climates to vary less in longitude than in latitude we consider the grid points in an ellipse (rather than a circle) with a major axis trending east-west. This modified ellipse-based distance is

(14)a=(1−w)d2+w(R|Δϕ|)2

where *d* is the distance, *R* is the radius of the earth, Δ*ϕ* is the latitudinal difference between the grid points and *w* is a weighting that determines the deviation from circular (*w*=0.75). Let *ε* be the grid spacing in latitude. We incrementally increase the ‘search *a*’ (=*a*_max_) by *ε* starting from 1.5*ε*. When there are land grid points within *a*_max_, we average these grid points together with an inverse-distance weighting 1/*a* to get the extrapolated terrestrial paleoclimates for the now-submerged target grid cell. Note that these extrapolated estimates of paleoclimates for now-submerged grid cells are likely to be more prone to systematic bias and higher uncertainty, so caution is warranted when making ecological or other inferences for now-submerged grid cells.

No special consideration was given to ice-covered surfaces. However, the Lutz *et al.*^63^ scheme has evaporation (AET) only when there is liquid water (rain or snow melt), so AET is zero over most ice-covered grid cells.

### Extracting century-scale averages from the decadal data

These downscaled datasets were originally developed for use with late-Quaternary fossil pollen records. Because of uncertainties in radiocarbon dates and other age controls, and because dating quality varies widely over paleoecological records that have been collected over the last 50 years, mapped syntheses of late-Quaternary paleoecological data typically have a maximum temporal resolution of ca. 500 years^66^, although the dating for individual records can be much more precise (10^1^ to 10^2^ years). Hence, for use with the synthesized fossil data, we extracted century-scale climatic averages from the paleoclimatic simulations.

Century-scale averages were calculated at 500 year intervals by averaging climate variables for all decades within 100 years of the target date. For example, for 0.5 ka BP, we averaged climatic values for 0.4 to 0.6 ka BP). The one exception is the 0 ka BP time window (0 ka BP is set to 1950 AD) where this interval is truncated depending on when the paleoclimatic simulation ended, producing an average spanning 1850 to 1990 AD for CCSM3 and 1850 to 1950 AD for ECBilt.

For each century-scale time window, we calculated several summary annual statistics. Statistics were first computed for individual decades, then averaged across all decades in the 200-year window. The statistics computed were annual sum for GDD, AET, PET, and precipitation and annual average for minimum and maximum daily temperature. We also computed estimates of variability, using the coefficient of variation for AET, PET, and precipitation. Because the coefficient of variation can be meaningless for data in an interval scale, we used the standard deviation for temperature related variables (GDD, minimum daily temperature, and maximum daily temperature). We also derived two indices of water availability: Evapotranspiration Ratio (ETR; AET/PET) and Water Deficit Index (WDI; PET—precipitation)^67,68^. Finally, we computed the highest and lowest monthly and seasonal (quarterly) values of each variable.

### Downscaling the IPCC AR5 simulations

The downscaling method for the IPCC AR5 simulations for the late 20th and 21st century closely followed the paleoclimatic simulations. However, several of the customized methods developed to work with the paleoclimatic simulations were unnecessary, because all IPCC AR5 variables were available as monthly values for each year, rather than decadal averages of monthly or seasonal values. Data were obtained from the Program for Climate Model Diagnosis and Intercomparison (PCMDI) website (http://cmip-pcmdi.llnl.gov/cmip5/data_portal.html).

Simulations for 12 models (Table 2) were downscaled with three simulations per model: Historical, RCP4.5, RCP8.5. The historical scenario covers 1950–2005 AD. The two future scenarios (RCP4.5 and RCP8.5) are from 2006 to 2100. All downscaled climate variables are available as monthly values for every year. Summary statistics were calculated as for the century-scale averages, but were calculated for 1950–2005 average in the historical dataset and for 20-year averages at 10-year intervals (2011–2030, 2021–2040, …, 2081–2100 AD) in the two climate scenarios (i.e., RCP4.5 and RCP8.5), instead of 200-year windows centred on 500-year intervals. This use of 20-year averages spaced 10 years apart creates interdependencies in the decadal averages (to avoid this, use decadal averages from every other decade), but enables calculation of climate means and other statistics that were not overly influenced by low sample size and interannual climate variability. Users wishing to construct alternate averages and summary statistics can make use of the monthly data available in the NetCDF files (Table 1 (available online only)).

### Code availability

All scripts used to develop the raster files with the centennial-scale and decadal-scale summaries are available on GitHub at the following repository: https://github.com/fitzLab-AL/climateSims21kto2100.

## Data Records

All datasets have been deposited with the Dryad Digital Repository (Data Citation 1). See Table 1 (available online only) for a full list of all datasets, associated metadata, and DOIs.

## Technical Validation

### Seasonal to monthly interpolation

#### Temperature

**Figure 3:**
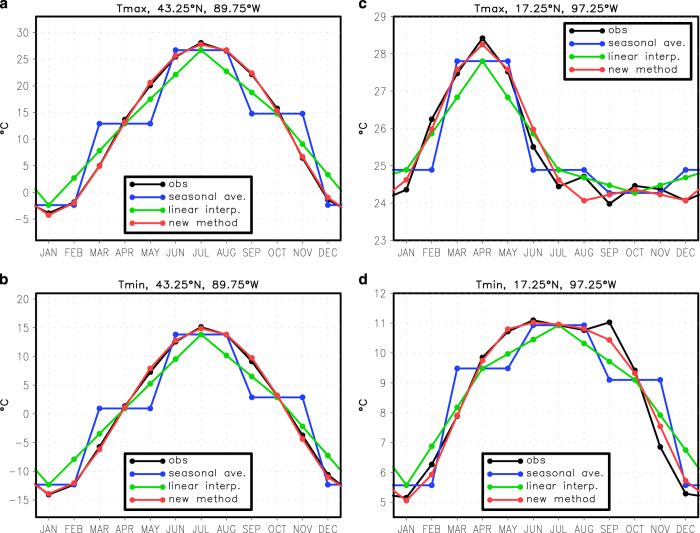
Assessment of the process used to predict the monthly annual cycle from the seasonal annual cycle for temperature. This plot shows maximum or minimum temperature annual cycle for Madison, WI ((**a**) and (**b**)) and a point northwest of Oaxaca, Mexico ((**c**) and (**d**)). The observed monthly annual cycle is in black. The observed monthly annual cycle averaged together into seasonal means is in blue. The monthly annual cycle estimated from the seasonal means by simple linear interpolation is in green. The monthly annual cycle estimated from the seasonal means by the new method described in the text is in red.

We assess the performance of the method used to predict the monthly annual cycle from the seasonal annual cycle by comparing the observed seasonally averaged cycle, the monthly annual cycle estimated from the linearly interpolated seasonal cycle, and the results from the new method described above. Both the maximum and minimum temperature are shown for a point near Madison, WI ((a) and (b)) and a point with a very different maximum temperature annual cycle ((c) and (d), northwest of Oaxaca, Mexico) (Fig. 3). For Madison the method works very well. For the Mexico location, the method also works reasonably well although the method does not pick out the higher-frequency variations in the annual cycle (in this case, some of the high-frequency variations may be sampling noise).

#### Precipitation

**Figure 4:**
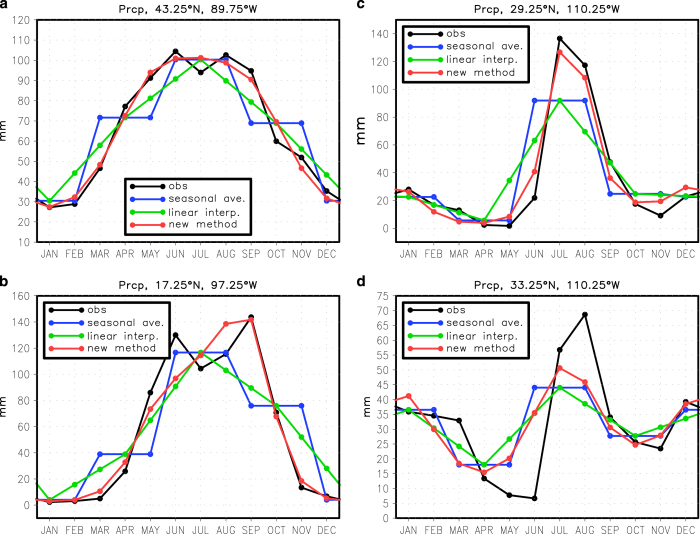
Estimating the monthly annual cycle from the seasonal annual cycle for precipitation. Shown is the precipitation annual cycle for (**a**) Madison, WI, (**b**) a point northwest of Oaxaca, Mexico, (**c**) a point in northwest Mexico, and (**d**) a point 4° north in Arizona. The observed monthly annual cycle is in black. The observed monthly annual cycle averaged together into seasonal means is in blue. The monthly annual cycle estimated from the seasonal means by linear interpolation is in green. The monthly annual cycle estimated from the seasonal means by the new method described in the text is in red.

We also assess the performance of these methods for deriving the monthly precipitation from the seasonal precipitation data (Fig. 4). The new method is certainly better than standard methods but sometimes there is simply not enough information in the seasonal averages to reproduce the high frequency components of the monthly annual cycle. In some cases, the new method does surprisingly well despite the fact there are strong changes in adjacent months. For example, the method captures much of the sudden onset of the North American monsoon in July (Fig. 4c). For a very similar situation a bit further north where winter precipitation is more pronounced, however, the method does not reproduce the abrupt onset of the monsoon. Hence, we recommend some caution and critical judgment when using the monthly precipitation data; these data are not well suited for capturing precipitation extremes and abrupt intra-annual shifts in rainfall. To avoid these limitations, we recommend that public archives of climate simulations store data at daily or monthly resolution, rather than seasonal resolution.

### Growing degree days: effect of using estimators of daily data

**Figure 5:**
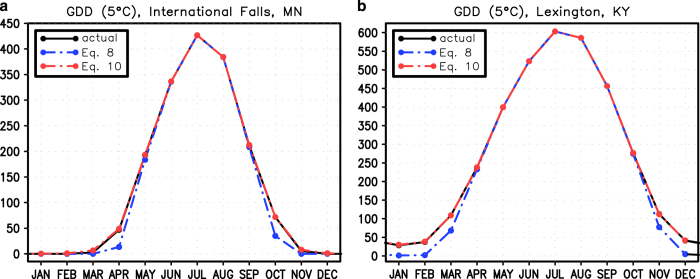
Estimating the monthly annual cycle of GDD base 5° using the monthly mean temperature only(blue) and the monthly mean temperature and the daily standard deviation (red). The actual GDD is in black. (**a**) International Falls, MN, (**b**) Lexington, KY.

We assess the effectiveness of using estimators of daily standard deviations in temperature to calculate GDD (Fig. 5). During the warm months both the standard approach (simply subtracting monthly temperatures from a base temperature (T_0_), equation 8) and the inclusion of estimates of daily standard deviations with the monthly data (equation 10) closely approximate the actual GDD. In the cool parts of the year, however, equation 10 does a much better job of estimating actual GDD.

### Cross-correlation structure in downscaled variables

Downscaling will debias each individual variable, but it does not necessarily preserve realistic correlations among climate variables. A biased correlation structure among downscaled climate variables can bias the calculation of derived variables such as PET and AET, or bias ecological simulations based on these climate datasets. Here, we compare the correlation structure in observations from the observed CRU dataset versus the correlation structure in monthly anomalies in the downscaled CMIP5 historical simulations. Because some variables, such as wind speed, are only available as climatologies, this correlation analysis is restricted to precipitation, mean maximum and minimum daily temperature, and vapour pressure.

**Figure 6:**
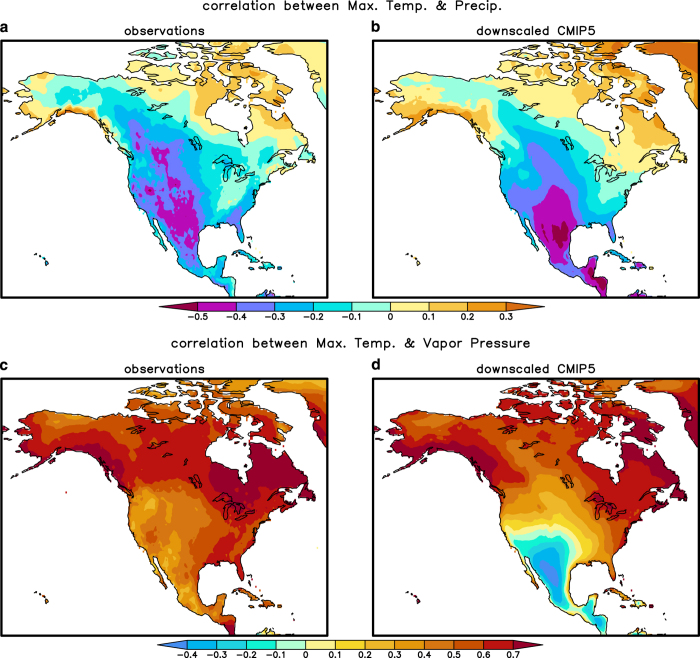
Maps of the temporal correlation between monthly anomalies for selected climate variables, to check whether the downscaled dataset has preserved the correlational structure in observational data. (**a**) Observed correlation between maximum temperature and precipitation (1950–2005). (**b**) As in (**a**) but for the downscaled CMIP5 data. This is the mean correlation averaged over 12 climate models. (**c**,**d**) As (**a**,**b**) but for maximum temperature and vapour pressure. The cross-correlation structure between temperature and precipitation is generally well-preserved, while the cross-correlation structure is less well preserved for vapour pressure.

The correlation between precipitation and temperature variables tends to match well between observations and the downscaled historical simulations. For example, the downscaling generally captures the correlation between maximum temperature and precipitation (Fig. 6a,b). The other correlations among precipitation and minimum and maximum temperature are similarly well captured. Correlation structure, however, is not well preserved between maximum temperature and vapour pressure (Fig. 6c,d) and precipitation and vapour pressure (Fig. 6e,f). The main discrepancies are in Mexico and the southwestern U.S and these discrepancies are also present in the raw climate model data. Diagnosing the reasons for these biases is beyond the scope of this research, but for maximum temperature and vapour pressure, we hypothesize that air temperature in the CMIP5 models is coupled too tightly to the land model, such that dry soil anomalies (which are correlated with vapour pressure) have too strong an effect on maximum temperature, via changes in sensible and latent heating. In actuality, temperature and vapour pressure likely are more controlled by atmospheric advection from remote locations. For precipitation and vapour pressure, perhaps the convection schemes in the CMIP5 models are too sensitive to boundary layer humidity as opposed to humidity in the atmosphere above the boundary layer, e.g. ref. 69.

**Figure 7:**
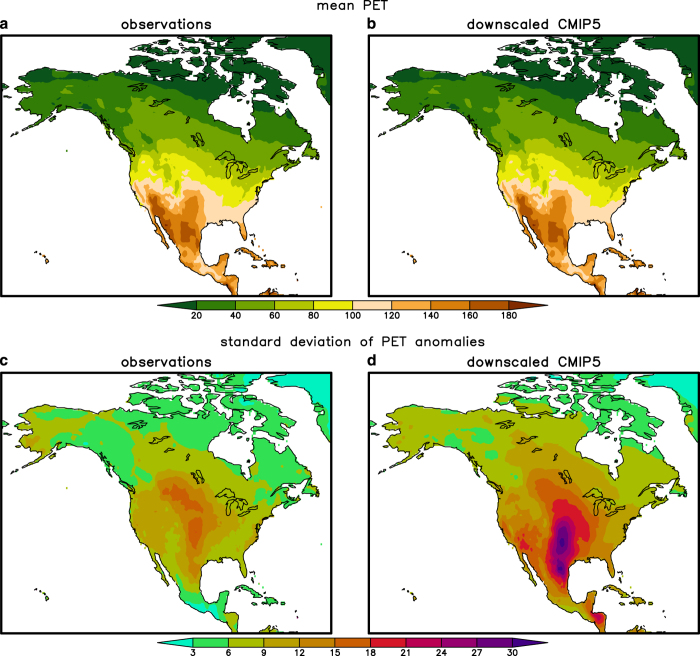
Comparison of observed and downscaled simulated potential evapotranspiration (PET), represented as annual averages of monthly data. (**a**) PET derived from observed variables such as temperature and vapour pressure. (**b**) As in (**a**) but for an average of 12 downscaled CMIP5 models. (**c**) Standard deviation of PET anomalies calculated from observational data. (**d**) As in (**c**) but for the downscaled CMIP5 models.

**Figure 8:**
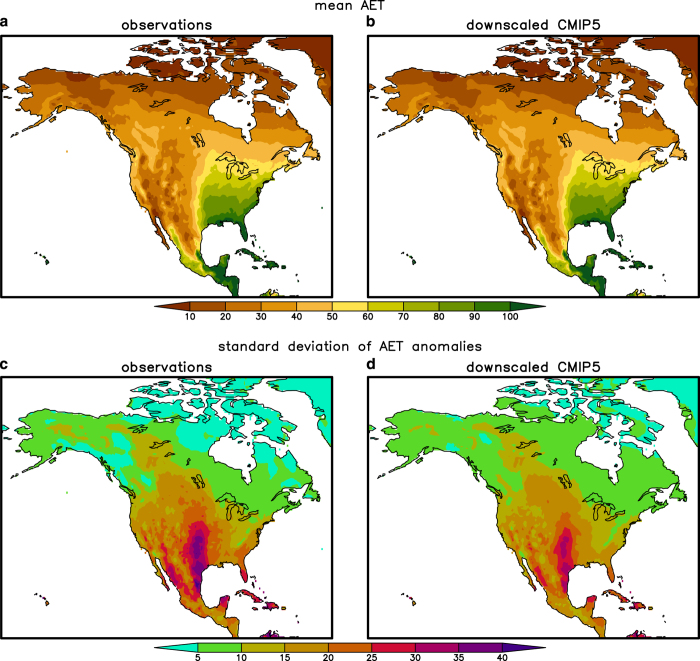
As Fig. 7, but for actual evapotranspiration (AET). (**a**) AET derived from observed variables such as temperature and vapour pressure. (**b**) As in (**a**) but for an average of 12 downscaled CMIP5 models. (**c**) Standard deviation of AET anomalies calculated from observational data. (**d**) As in (**c**) but for the downscaled CMIP5 models.

We also checked to see whether correlation biases involving vapour pressure affect derived variables such as PET and AET (Figs 7 and 8). Both mean PET and AET agree well between observations and the downscaled simulations (Figs 7 and 8) and do not appear to be affected by these correlation biases. The good fit likely emerges because the total variation in AET and PET is a combination of the mean annual cycle and the monthly anomalies from the mean annual cycle. Apparently the mean annual cycle dominates and therefore the mean PET is well simulated. However, the correlation biases do impact the standard deviation of the PET anomalies from the mean annual cycle (Fig. 7c,d). The variance in downscaled PET is too large because the correlation between maximum temperature (*T*_max_) and vapour pressure (*e*_*s*_) is too small or even negative. Therefore the difference between the saturation vapour pressure (*e*_sat_) at temperature *T*_max_ (i.e., *e*_sat_(*T*_max_)) and *e*_*s*_ varies more than if *e*_sat_ (*T*_max_) and *e*_*s*_ were highly correlated. Because one of the two terms in PET is proportional to *e*_sat_ (*T*_max_)−*e*_*s*_, PET also has too much variance.

Like PET, mean AET is also very well reproduced in the downscaling (Fig. 8). Unlike PET, however, the standard deviation for AET is also quite good in the downscaling and, moreover, the bias tends to have the opposite sign. This behaviour is due to the competing effects of the maximum temperature/vapour pressure bias and the precipitation/vapour pressure bias. For example, in the southwest U.S., increased vapour pressure leads to decreased PET (and likewise AET) via the former bias, but leads to increased precipitation, wetter soil and increased AET via the latter bias. Because the main biases are all related to vapour pressure, for future downscaling efforts, we recommend parameterizing vapour pressure (or dew point temperature) in terms of temperature and/or precipitation instead of downscaling it directly.

## Usage Notes

### General considerations

In both paleoclimatic simulations, the timescale is expressed as time before present, where ‘present’ follows radiocarbon dating conventions and is defined as 0 ka BP (1950). Decade 0 is defined as from 1st January 1951 to 31st December 1960. CCSM3 data extends from decade −2200 to +3, which means it ends on December 31st, 1990, whereas ECBilt-CLIO goes from −2100 to −1, which means this simulation ends on December 31st, 1950.

Because debiasing and downscaling is imperfect, whenever possible, comparative analyses among climatic simulations should rely upon simulations from the same model or rely upon ensembles based on the same sets of models (e.g.,, the ACCESS3-1 simulations for the historical and the RCP4.5 scenarios). Similarly, future and paleoclimatic simulations should be compared to the downscaled climate simulations from the historical IPCC AR5 simulation or the available decades for the last centennial from the paleoclimatic simulations (1850–1990 for CCSM3 and 1850–1950 for ECBilt-CLIO), rather than to observational datasets for the 20th and 21st century, including to the original CRU dataset. Some model switching is unavoidable when going from the paleoclimatic to future climate simulations. However, by using standard observational baselines and methods we have minimized the effect of this switching.

Note that these paleoclimatic simulations contain no estimates of uncertainty, nor do they assess whether there is systematic bias between the paleoclimatic simulations and inferences based on paleoclimatic proxies. Paleoclimatic model simulations carry inherent uncertainty both in model structure (how processes are represented in models) and parameterization (the values of parameters used within the model). There is a rich literature in paleoclimatic data-model comparisons and syntheses, particularly for the Paleoclimatic Intercomparison Modeling Project, e.g. (refs 39,70–72) and for the SynTrace simulations that are downscaled here^41,42,46,73,74^. Users are encouraged to refer to this literature to check for known data-model discrepancies before beginning work with the paleoclimatic simulations. A critical and on-going need is to understand how uncertainties in model choice, structure, and parameterization propagate to uncertainties in paleoclimatic simulations.

### Primary and secondary variables (NetCDF)

All primary and secondary variables are stored as NetCDF files (see Table 1 (available online only) for file names and contents). For the paleoclimatic simulations (CCSM3 and EC-BILT), the individual netCDFs for each variable are archived separately (Table 1 (available online only)). For the 21st-century simulations, the individual netCDFs are organized into a directory structure as follows: cmip5\[emissions scenario]\[ESM model name]\[variable name.nc]. For example, cmip5\rcp45\ACCESS1-3\ET.nc holds the evapotranspiration simulations produced by the ACCESS1-3 ESM (Table 2) for emissions scenario RCP4.5.

In the NetCDF files, the data have been packed into short integers (each requiring 2 bytes) instead of real numbers (requiring 4 bytes) to save space. One must unpack that data to get the correct floating point representation of the data. Each netCDF variable that has been packed has an add_offset and scale_factor attribute associated with it. The formula to unpack the data is:

unpacked value=add_offset+((packed value) * scale_factor )

For more information see: http://www.unidata.ucar.edu/software/netcdf/docs/attribute_conventions.html.

The ‘missing_value’ attribute in the NetCDF data is set to −32768. Only grid points outside the downscaling domain are given the missing data value. The ncdf4 package in R (https://cran.r-project.org/web/packages/ncdf4/index.html) allows unpacking of these files with automatic correction for the offset, scale factor or the missing values.

### Centennial and decadal variables (geotiffs)

The centennial- and decadal-scale summaries produced for input into ecological models are stored as geotiffs (TIFF format, .tif). Each raster holds a unique combination of climate variable, ESM, and time period, producing a large number of individual rasters. The rasters are zipped into compressed files organized by paleoclimatic simulation and CMIP5 emissions scenario (Table 1 (available online only)). Rasters are organized into a directory structure that differs slightly for the paleoclimatic and CMIP5 simulations. For the paleoclimatic rasters, the directory structure is [model name]\[time period]\[variable name].tif. For the 21st-century rasters, the directory structure is [CMIP5 scenario name]\[ESM model name]\[time period]\[variable name].tif. For example, RCP4.5\ACCESS1-3\2100\mo-lwr-TMIN.tif stores the lower monthly value of minimum daily temperatures, expected for year 2100 for the ACCESS1-3 ESM simulation under the CMIP5 RCP4.5 emissions scenario (Table 3). The naming conventions for variables and raster files are described in Table 3.

## Additional Information

**How to cite this article:** Lorenz, D. J. *et al.* Downscaled and debiased climate simulations for North America from 21,000 years ago to 2100AD. *Sci. Data* 3:160048 doi: 10.1038/sdata.2016.48 (2016).

## Supplementary Material



## Figures and Tables

**Table 1:** List and metadata for all data files archived at Data Dryad and produced during debiasing, downscaling, and summarization

**Data Type**	**Climate Model**	**Variable(s)**	**Units**	**Dimensions**	**Spatial Resolution**	**Temporal Range**	**Temporal Resolution**	**Filename**	**Format**	**File Size**	**DOI**
NetCDF	CCSM3	mean minimum daily temperature, mean maximum daily temperature	C	250 lon x 140 lat	0.5 degrees	22,000 years before 1950 AD to 1990 AD	decadal means of months	ccsm3_22-0k_temp.nc	NetCDF	1,182 GB	http://dx.doi.org/10.5061/dryad.1597g
NetCDF	CCSM3	potential evapotranspiration, actual evapotranspiration	mm	250 lon x 140 lat	0.5 degrees	22,000 years before 1950 AD to 1990 AD	decadal means of months	ccsm3_22-0k_et.nc	NetCDF	738 GB	http://dx.doi.org/10.5061/dryad.1597g
NetCDF	CCSM3	mean total monthly precipitation	mm	250 lon x 140 lat	0.5 degrees	22,000 years before 1950 AD to 1990 AD	decadal means of months	ccsm3_22-0k_prcp.nc	NetCDF	544 GB	http://dx.doi.org/10.5061/dryad.1597g
NetCDF	CCSM3	growing degree days base 5C, growing degree days base 0C	days C	250 lon x 140 lat	0.5 degrees	22,000 years before 1950 AD to 1990 AD	decadal means of months	ccsm3_22-0k_gdd.nc	NetCDF	835 GB	http://dx.doi.org/10.5061/dryad.1597g
NetCDF	CCSM3	mean monthly net downward longwave radiation at surface	W m^-2	250 lon x 140 lat	0.5 degrees	22,000 years before 1950 AD to 1990 AD	decadal means of months	ccsm3_22-0k_lwn.nc	NetCDF	621 GB	http://dx.doi.org/10.5061/dryad.1597g
NetCDF	CCSM3	mean monthly downward shortwave radiation at surface	W m^-2	250 lon x 140 lat	0.5 degrees	22,000 years before 1950 AD to 1990 AD	decadal means of months	ccsm3_22-0k_swd.nc	NetCDF	492 GB	http://dx.doi.org/10.5061/dryad.1597g
NetCDF	CCSM3	mean monthly upward shortwave radiation at surface	W m^-2	250 lon x 140 lat	0.5 degrees	22,000 years before 1950 AD to 1990 AD	decadal means of months	ccsm3_22-0k_swu.nc	NetCDF	488 GB	http://dx.doi.org/10.5061/dryad.1597g
NetCDF	CCSM3	mean monthly water vapor pressure	hPa	250 lon x 140 lat	0.5 degrees	22,000 years before 1950 AD to 1990 AD	decadal means of months	ccsm3_22-0k_vap.nc	NetCDF	498 GB	http://dx.doi.org/10.5061/dryad.1597g
NetCDF	CCSM3	mean monthly wind speed	m s^-1	250 lon x 140 lat	0.5 degrees	22,000 years before 1950 AD to 1990 AD	decadal means of months	ccsm3_22-0k_wnd.nc	NetCDF	483 GB	http://dx.doi.org/10.5061/dryad.1597g
NetCDF	EC-BILT	mean minimum daily temperature, mean maximum daily temperature	C	250 lon x 140 lat	0.5 degrees	21,000 years before 1950 AD to 1950 AD	decadal means of months	ecbilt_21-0k_temp.nc	NetCDF	1,117 GB	http://dx.doi.org/10.5061/dryad.1597g
NetCDF	EC-BILT	potential evapotranspiration, actual evapotranspiration	mm	250 lon x 140 lat	0.5 degrees	21,000 years before 1950 AD to 1950 AD	decadal means of months	ecbilt_21-0k_et.nc	NetCDF	739 GB	http://dx.doi.org/10.5061/dryad.1597g
NetCDF	EC-BILT	mean total monthly precipitation	mm	250 lon x 140 lat	0.5 degrees	21,000 years before 1950 AD to 1950 AD	decadal means of months	ecbilt_21-0k_prcp.nc	NetCDF	522 GB	http://dx.doi.org/10.5061/dryad.1597g
NetCDF	EC-BILT	growing degree days base 5C, growing degree days base 0C	days C	250 lon x 140 lat	0.5 degrees	21,000 years before 1950 AD to 1950 AD	decadal means of months	ecbilt_21-0k_gdd.nc	NetCDF	835 GB	http://dx.doi.org/10.5061/dryad.1597g
NetCDF	EC-BILT	mean monthly net downward longwave radiation at surface	W m^-2	250 lon x 140 lat	0.5 degrees	21,000 years before 1950 AD to 1950 AD	decadal means of months	ecbilt_21-0k_lwn.nc	NetCDF	587 GB	http://dx.doi.org/10.5061/dryad.1597g
NetCDF	EC-BILT	mean monthly downward shortwave radiation at surface	W m^-2	250 lon x 140 lat	0.5 degrees	21,000 years before 1950 AD to 1950 AD	decadal means of months	ecbilt_21-0k_swd.nc	NetCDF	469 GB	http://dx.doi.org/10.5061/dryad.1597g
NetCDF	EC-BILT	mean monthly upward shortwave radiation at surface	W m^-2	250 lon x 140 lat	0.5 degrees	21,000 years before 1950 AD to 1950 AD	decadal means of months	ecbilt_21-0k_swu.nc	NetCDF	457 GB	http://dx.doi.org/10.5061/dryad.1597g
NetCDF	EC-BILT	mean monthly water vapor pressure	hPa	250 lon x 140 lat	0.5 degrees	21,000 years before 1950 AD to 1950 AD	decadal means of months	ecbilt_21-0k_vap.nc	NetCDF	475 GB	http://dx.doi.org/10.5061/dryad.1597g
NetCDF	12 CMIP5 models, historical (2 files)	All variables	--	250 lon x 140 lat	0.5 degrees	1950 AD to 2005 AD	decadal intervals, monthly	cmip5_hist_all_1.zip cmip5_hist_all_2.zip	NetCDF	920 GB 920 GB	http://dx.doi.org/10.5061/dryad.1597g
NetCDF	12 CMIP5 models, RCP4.5 (3 files)	All variables	--	250 lon x 140 lat	0.5 degrees	2010 AD to 2100 AD	decadal intervals, monthly	cmip5_rcp45_all_1.zip cmip5_rcp45_all_2.zip cmip5_rcp45_all_3.zip	NetCDF	1,062 GB 1,060 GB 1,056 GB	http://dx.doi.org/10.5061/dryad.1597g
NetCDF	12 CMIP5 models, RCP8.5 (3 files)	All variables	--	250 lon x 140 lat	0.5 degrees	2010 AD to 2100 AD	monthly	cmip5_rcp85_all_1.zip cmip5_rcp85_all_2.zip cmip5_rcp85_all_3.zip	NetCDF	1,069 GB 1,069 GB 1,064 GB	http://dx.doi.org/10.5061/dryad.1597g
GeoTIF summaries	CCSM3	All—see Table 3	--	250 lon x 140 lat	0.5 degrees	22,000 years before 1950 AD to 1990 AD	200-year means, spaced 500 years apart, of monthly, seasonal, and annual variables	ccsm3_22-0k_all_tifs.zip	GEOTIFF	0.25 GB	http://dx.doi.org/10.5061/dryad.1597g
GeoTIF summaries	EC-BILT	All—see Table 3	--	250 lon x 140 lat	0.5 degrees	21,000 years before 1950 AD to 1950 AD	200-year means, spaced 500 years apart, of monthly, seasonal, and annual variables	ecbilt_21-0k_all_tifs.zip	GEOTIFF	0.24 GB	http://dx.doi.org/10.5061/dryad.1597g
GeoTIF summaries	12 CMIP5 models, historical	All—see Table 3	--	250 lon x 140 lat	0.5 degrees	1950 AD to 2005 AD	55-year mean climatology for late 20th / early 21st century	cmip5_hist_1950-2005_all_tifs.zip	GEOTIFF	0.06 GB	http://dx.doi.org/10.5061/dryad.1597g
GeoTIF summaries	12 CMIP5 models, RCP4.5	All—see Table 3	--	250 lon x 140 lat	0.5 degrees	2006 AD to 2100 AD	Decadal intervals, of seasonal, and annual variables	cmip5_rcp4.5_2006-2100_all_tifs.zip	GEOTIFF	0.49 GB	http://dx.doi.org/10.5061/dryad.1597g
GeoTIF summaries	12 CMIP5 models, RCP8.5	All—see Table 3	--	250 lon x 140 lat	0.5 degrees	2006 AD to 2100 AD	Decadal intervals, of seasonal, and annual variables	cmip5_rcp8.5_2006-2100_all_tifs.zip	GEOTIFF	0.50 GB	http://dx.doi.org/10.5061/dryad.1597g

**Table 2:** CMIP5 climate models used for 21st-century projections (RCP4.5 and RCP8.5).

**Modeling Center or Group**	**Model Name**
Commonwealth Scientific and Industrial Research Organization (CSIRO) and Bureau of Meteorology (BOM), Australia	ACCESS1.3
Canadian Centre for Climate Modelling and Analysis	CanESM2
Community Earth System Model Contributors	CESM1(CAM5)
Centre National de Recherches Météorologiques / Centre Européen de Recherche et Formation Avancée en Calcul Scientifique	CNRM-CM5
Commonwealth Scientific and Industrial Research Organization in collaboration with Queensland Climate Change Centre of Excellence	CSIRO-Mk3.6.0
NOAA Geophysical Fluid Dynamics Laboratory	GFDL-CM3
NASA Goddard Institute for Space Studies	GISS-E2-R
Met Office Hadley Centre (additional HadGEM2-ES realizations contributed by Instituto Nacional de Pesquisas Espaciais)	HadGEM2-ES
Institute for Numerical Mathematics	INM-CM4
Institut Pierre-Simon Laplace	IPSL-CM5A-MR
Atmosphere and Ocean Research Institute (The University of Tokyo), National Institute for Environmental Studies, and Japan Agency for Marine-Earth Science and Technology	MIROC5
Meteorological Research Institute	MRI-CGCM3

**Table 3:** Naming convention for variable names in raster files generated for ecological modeling with centennial and decadal summaries.

**Variable**	**Annual**		**Quarterly**		**Monthly**	
	**Summaries**	**Variability**	**Min**	**Max**	**Min**	**Max**
Tmin	an-avg-TMIN	an-sd-TMIN	qt-lwr-TMIN		mo-lwr-TMIN	
Tmax	an-avg-TMAX	an-sd-TMAX		qt-hgr-TMAX		mo-hgr-TMAX
Prec	an-sum-PRCP	an-cv-PRCP	qt-lwr-PRCP	qt-hgr-PRCP	mo-lwr-PRCP	mo-hgr-PRCP
GDD	an-sum-GDD	an-sd-GDD	qt-lwr-GDD	qt-hgr-GDD	mo-lwr-GDD	mo-hgr-GDD
AET	an-sum-AET	an-cv-AET	qt-lwr-AET	qt-hgr-AET	mo-lwr-AET	mo-hgr-AET
PET	an-sum-PET	an-cv-PET	qt-lwr-PET	qt-hgr-PET	mo-lwr-PET	mo-hgr-PET
ETR (AET/PET)	an-avg-ETR	an-cv-ETR	qt-lwr-ETR	qt-hgr-ETR	mo-lwr-ETR	mo-hgr-ETR
WDI (PET—Prec)	an-sum-WDI	an-cv-WDI	qt-lwr-WDI	qt-hgr-WDI	mo-lwr-WDI	mo-hgr-WDI
